# Pediatric Dysfunctional Breathing: Proposed Components, Mechanisms, Diagnosis, and Management

**DOI:** 10.3389/fped.2020.00379

**Published:** 2020-07-16

**Authors:** Nicki Barker, Ravi Thevasagayam, Kelechi Ugonna, Jane Kirkby

**Affiliations:** Sheffield Children's NHS Foundation Trust, Sheffield, United Kingdom

**Keywords:** dysfunctional breathing, breathing pattern disorders, inducible laryngeal obstruction, pediatrics, exercise induced dyspnoea, vocal cord dysfunction

## Abstract

Dysfunctional breathing (DB) is an overarching term describing deviations in the normal biomechanical patterns of breathing which have a significant impact on quality of life, performance and functioning. Whilst it occurs in both children and adults, this article focuses specifically on children. DB can be viewed as having two components; breathing pattern disorder (BPD) and inducible laryngeal obstruction (ILO). They can be considered in isolation, however, are intricately related and often co-exist. When both are suspected, we propose both BPD and ILO be investigated within an all-encompassing multi-disciplinary dysfunctional breathing clinic. The MDT clinic can diagnose DB through expert history taking and a choice of appropriate tests/examinations which may include spirometry, breathing pattern analysis, exercise testing and laryngoscopic examination. Use of the proposed algorithm presented in this article will aid decision making regarding choosing the most appropriate tests and understanding the diagnostic implications of these tests. The most common symptoms of DB are shortness of breath and chest discomfort, often during exercise. Patients with DB typically present with normal spirometry and an altered breathing pattern at rest which is amplified during exercise. In pediatric ILO, abnormalities of the upper airway such as cobblestoning are commonly seen followed by abnormal activity of the upper airway structures provoked by exercise. This may be associated with a varying degree of stridor. The symptoms, however, are often misdiagnosed as asthma and the picture can be further complicated by the common co-presentation of DB and asthma. Associated conditions such as asthma, extra-esophageal reflux, rhinitis, and allergy must be treated appropriately and well controlled before any directed therapy for DB can be started if therapy is to be successful. DB in pediatrics is commonly treated with a course of non-pharmaceutical therapy. The therapy is provided by an experienced physiotherapist, speech and language therapist or psychologist depending on the dominant features of the DB presentation (i.e., BPD or ILO in combination or in isolation) and some patients will benefit from input from more than one of these disciplines. An individualized treatment program based on expert assessment and personalized goals will result in a return to normal function with reoccurrence being rare.

## Introduction

Dysfunctional breathing (DB) is an overarching term describing deviations in the normal biomechanical patterns of breathing that result in intermittent or chronic symptoms (1). It is a respiratory condition which affects both children and adults, and although more comprehensively described in adults, the impact of the condition is no less significant in children. DB has been shown to have a greater impact on health-related quality of life than asthma (2, 3). In this article, we will describe the features and sub-classifications of DB in children, the proposed mechanisms of it, and how to diagnose and manage the condition in the pediatric setting.

## Methods

Pubmed was searched using the terms dysfunctional breathing, breathing pattern disorders, inducible laryngeal obstruction, vocal cord dysfunction, pediatrics, and exercise testing. Further literature was sourced from the reference lists of identified articles and from conference proceedings.

### Dysfunctional Breathing

**Figure 1:**
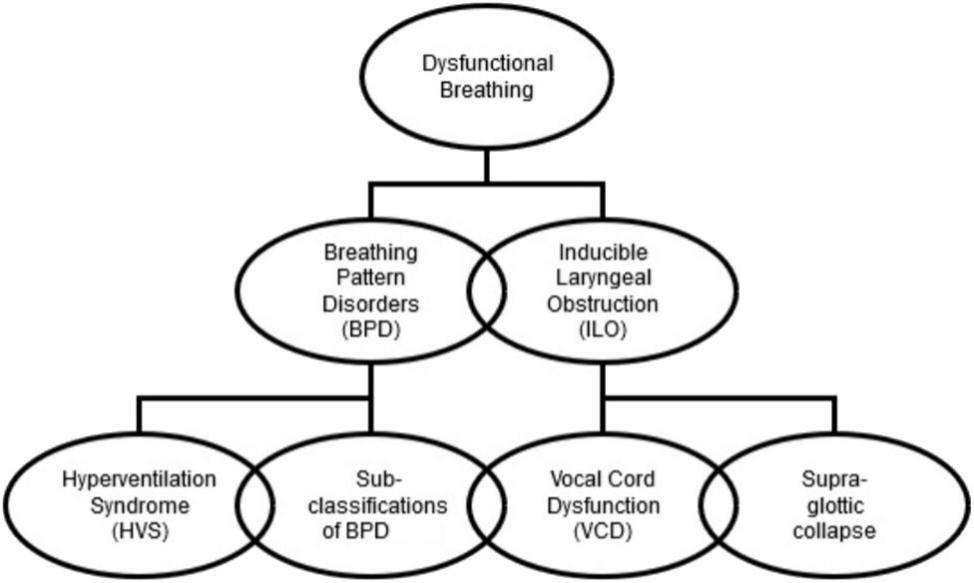
Organizational chart showing the components and sub-components of dysfunctional breathing.

Dysfunctional breathing is a term describing changes in breathing pattern that result most commonly in dyspnoea (4). Dysfunctional breathing can be viewed in two component parts; a breathing pattern component and an upper airway component. Whilst dysfunction with each component may occur independently of the other, they can be intricately related and may co-exist (5). These components are commonly referred to as breathing pattern disorder (BPD) and inducible laryngeal obstruction (ILO) or EILO where the laryngeal obstruction is induced by exercise. The individual presentation of DB is influenced by the proportions in which these two components are present. Detailed assessment in an all-encompassing multi-disciplinary clinic is required to ascertain the makeup of the DB, such as whether it is predominantly caused by ILO, BPD or a combination of the two, enabling more targeted interventions. A proposed organizational chart is shown in Figure 1 which shows the sub-classifications of each component. These sub-classifications are described in more detail below.

### Prevalence

One of the great challenges of DB is the similarity of symptoms with other respiratory disorders, such as asthma and exercise induced bronchoconstriction (EIB), and the possibility of co-existence with these disorders. Given these similarities of presentation and co-existence, and the differing diagnostic pathways (6), defining the prevalence of DB amongst the general population is challenging, particularly as each sub-category is often reviewed separately and there are few data in children. For example, the prevalence of Hyperventilation Syndrome and Breathing Pattern Disorders in adult populations has been estimated between 6 and 10% by various studies (7–9), whereas due to a lack of standardized definitions and a lack of recognition of its importance, the prevalence of BPD in pediatric populations is unknown (10) and has not currently been reported by any single study. In a cross-sectional study of adolescents with exercise induced dyspnea 39.8% had EIB, 6% had EILO and 4.5% had both (11).

The prevalence of EILO in the general population of adolescence can also be difficult to define and varies depending on the population group studied. Olin et al. (12) quoted two European studies (11, 13) that suggested the prevalence across adolescence and young adults at around 5–8%, whereas Kolnes et al. (14) estimated the prevalence of EILO as 7% in the general adolescent population and 35% in athletes, and they described EILO being more common in athletes involved in high intensity interval exercises. Other studies have documented DB and specifically EILO being more common in females, and it being more notable in the Caucasian population, however much of the information comes from adult studies (12). Further research in the pediatric populations is required.

### The Mechanisms of Dysfunctional Breathing: A Breathing Pattern Component and/or an Upper Airway Component

#### Breathing Pattern Disorders (BPD)

A breathing pattern disorder is a form of dysfunctional breathing that occurs when the normal relaxed respiratory cycle is replaced either intermittently or habitually with abnormal breathing patterns (5). Attempts have been made to classify different types of abnormal breathing pattern in adult patients with five different patterns being identified; hyperventilation syndrome, periodic deep sighing, thoracic dominant breathing, forced abdominal expiration and thoraco-abdominal asynchrony (15). A similar study carried out with children and young people has yet to be performed. The Boulding group used recordings of quiet tidal breathing at rest followed by maximal expiration then inspiration, recorded as volume time graphs (apart from thoraco-abdominal asynchrony which used movement time graphs), to identify the five types of abnormal breathing patterns. The key features of each are as follows:

- Hyperventilation syndrome—Rapid respiratory rate with tidal breathing closer to inspiratory capacity.
- Periodic deep sighing—Erratic breathing pattern with difficulty coordinating the maximal expiratory and inspiratory maneuver.
- Thoracic dominant breathing—Large volume breaths with minimal inspiratory reserve capacity.
- Forced abdominal expiration—Tidal breathing occurs at low lung volumes with minimal expiratory reserve volume.
- Thoraco-abdominal asynchrony—Asynchrony between rib cage and abdominal movement.

In young people however there is overlap between types of pattern and much variation is seen depending on the circumstances the young person is in. Forced abdominal expiration is rarely seen in pediatric DB, potentially due to this pattern being more commonly associated with COPD (16). Factors that impact on the breathing pattern include stress (both internal and external) and anxiety, and BPD are commonly found in the self-driven person (17).

Patients diagnosed with BPD commonly report symptoms with exercise (17). The normal response to exercise is an increase in respiratory rate and volumes but in BPD exercise appears to exaggerate the sometimes subtly abnormal breathing patterns which occur at rest (10). Breathing patterns however can become so chaotic or rapid that the patient can experience feelings of shortness of breath (with or without chest discomfort) at rest. This is seen to have an impact on social functioning such as attending school. Where BPD occur at high levels of sport or performance, it may subsequently have a negative impact on future career choices.

#### Inducible Laryngeal Obstruction

Dysfunctional breathing may also have an upper airway component. Inducible Laryngeal obstruction (ILO) occurs when an event, situation or specific irritant causes the laryngeal structures to impede the passage of air in and out of the trachea. This appears to occur when the inspiratory phase of the respiratory cycle is out of synch with vocal cord movement, hence the vocal cords are partially or completely adducted at this time. It can also occur when there are other forms of supra-glottic collapse during inspiration, either anterior/epiglottic or posterior/supra arytenoid (6). There is often an inspiratory stridor and a feeling of dyspnoea which may also be associated with throat or chest discomfort.

**Figure 2:**
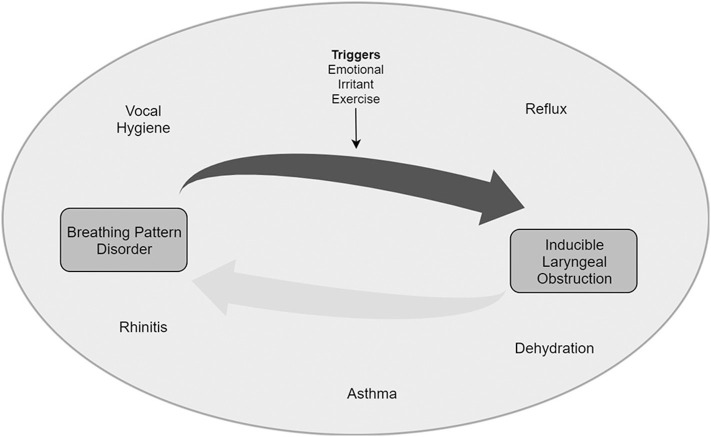
Proposed mechanism of progression and relationship between BPD and ILO.

The exact cause of Inducible Laryngeal Obstruction remains unknown, but it appears to be multifactorial (see Figure 2). The factors and the extent to which each plays a role in individual patients varies and creates a near unique clinical picture.

We hypothesize that there is a group of conditions which are co factors. They can be referred to as “enhancers.” These typically promote vocal cord closure, usually as a protective reflex in the first instance. The most common enhancers in pediatrics are extra-esophageal reflux (i.e., where reflux of gastric contents impacts beyond the esophagus), nasal obstruction and asthma (18).

1. Extra-esophageal reflux can be present with patients reporting few if any symptoms—so called “silent reflux,” however, on direct questioning many patients may have classical heartburn. Globus sensation may also be present. This feeling of a foreign body sensation in the throat can promote habitual throat clearing. This throat clearing may increase laryngeal irritability and sensitivity. There is good evidence for reflex esophageal mediated laryngeal closure caused by falls in pH (19).
2. Nasal obstruction promotes mouth breathing which may cause laryngeal dryness and increased irritability. This bypasses nasal breathing which normally promotes cordal opening. Postnasal drip secondary to nasal disease is also known to promote vocal cord closure (20).
3. Asthma commonly co-exists with ILO (21). A recent review by this group reported a pooled prevalence of 25% in adult patients with asthma and laryngeal obstruction. EIB may increase work of breathing, exacerbate BPD and anxiety, especially around potentially choking, and this may lead to ILO.

The enhancers appear to promote an environment in which a larynx is primed to closure if exposed to a stimulus that might not cause this in a non-primed larynx. The stimuli can be referred to as “triggers” or “inducers.” The commonest triggers in young people are exercise and emotional states, particularly anxiety where dysfunctional breathing is more common in chronic anxiety states (15). Another less common trigger is inhalation of an irritant smell.

The role of psychological well-being especially with anxiety states may often be seen in DB (22). This link has been so strong at various times that DB has been considered to be a somatisation illness—a clinical manifestation of mental illness (23). That said, the exact relationship is contentious especially regarding whether it is causative or a rational response to the sensation of choking. Consideration of mental state must be included in any proposed mechanism and assessment process. Whilst volitional movements of the vocal cords do occur (e.g., during vocalization, breath-holding, and cough), the opening and closure of the vocal cords in time with breathing is autonomic in nature and it may be that enhanced anxiety states disrupt autonomic control of vocal cord movements.

Dysfunctional breathing may be present at rest, but in a non-stressed state, adequate respiratory function can usually be maintained (15). In patients who have an enhancer (e.g., asthma, reflux, rhinitis, vocal hygiene, dehydration), we hypothesize that a state of laryngeal “hyper-responsiveness” can exist. In this “hyper-responsive” state a trigger (e.g., exercise, emotional, irritant etc.) may disrupt autonomic cordal opening. This impairs autonomic cordal function in time with breathing and in turn causes cordal closure during inspiration, and the symptoms the patients described. In this proposed mechanism, cordal closure is not truly paradoxical but in fact mistimed with regard to cordal position during phase of respiration.

Inspiration against a partially closed glottis may result in supraglottic collapse via the “Venturi effect” (based on the Bernoulli equation). In this proposed mechanism the supraglottic collapse seen and reported by other groups is secondary to cordal closure during inspiration. These problems may explain adequate airflow during rest but dyspnoea during stressed states such as exercise.

## Diagnosis

The challenges faced in diagnosing dysfunctional breathing often overshadow the need for a clear pathway for the management of young people with this condition. The pathway should start at the first suspicion of the involvement of DB and end with a resolution to the problem. A multi-disciplinary team approach is required to ensure that all aspects of the condition are investigated and addressed and that co-morbidities are effectively managed.

### Detailed History

A good history is essential in reaching a diagnosis. This should focus not only on the presenting features but also explore possible frequent co-morbidities like asthma, sinusitis, gastro-esophageal reflux and psychosocial issues.

Shortness of breath is the most common presenting complaint (4). It may be precipitated by exertion and is often worse with increasing intensity and duration of exercise, however it can occur at rest. It is also often made worse with anxiety, or anticipation. The symptom may also by provoked by various factors like singing, playing a musical instrument, panic and even environmental factors like specific aerosols or irritants (24).

The child may be able to specifically describe difficulty taking a breath in which may indicate more of an upper airway issue like ILO or getting a breath out which may indicate a lower airway issue such as asthma. In practice however, pin-pointing the period in the breathing cycle that is a problem is often not easy to discern for the child and cannot be considered entirely specific for determining the location of the problem.

Breathing may be noisy and there may be stridor present. This is more common with ILO, than in BPD alone. A cough and throat clearing can be present which is often associated with inflammation in the upper-airway and can be associated with sensitization of the vocal cords leading to paradoxical movement and ILO. Chest pain, tightness or discomfort is frequently reported and may be of biochemical or biomechanical cause or related to associated exercise induced bronchoconstriction or gastro-esophageal reflux. Patients sometimes refer to an obstruction or lump in the throat during the episode, or indicate they feel the obstruction in the level of the upper chest or throat.

As Gastro-esophageal reflux is a common co-morbidity (25), specific questions relating to this including the presence of heartburn, water brash, nausea and vomiting and high-risk eating habits like large meals close to bedtime should be asked. A pH study may be helpful, whilst remembering that it is still unclear what the diagnostic threshold is for extra-esophageal reflux. As sinusitis is a frequent comorbidity, symptoms such as rhinitis, nasal congestion and mouth breathing as well as frequent ear infections and deafness should also be sought (26). Asthma is also sometimes a co-morbidity but is also frequently confused for dysfunctional breathing. Features that relate to asthma like wheeze, di-urnal variation, specific triggers, interval symptoms and nocturnal cough should be sought. Anxiety is the most frequent psychosocial co-morbidity. Features of panic including palpitations, dizziness and hyperhidrosis should also be sought.

### Differential Diagnosis

**Figure 3:**
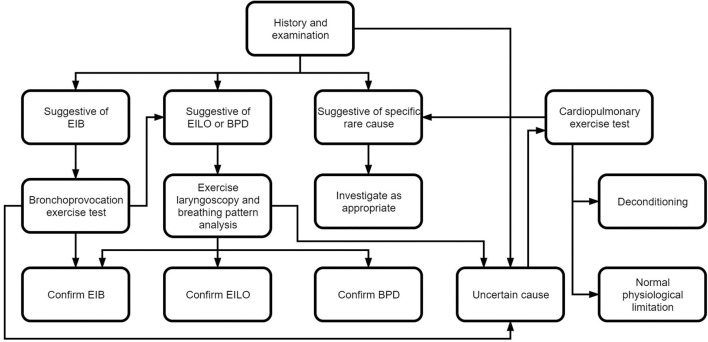
Diagnostic testing algorithm.

Dysfunctional breathing is associated with chronic dyspnea, therefore any other causes of chronic dyspnea should be considered and excluded (see Figure 3).

The most common cause of dyspnea in children is asthma and this is therefore one of the main differentials (27). As both conditions sometimes co-exist (28), it is important to ensure that any airway hyperactivity and bronchoconstriction is identified and treated. Features of the history suggestive of asthma are discussed above, spirometry, exhaled nitric oxide levels and bronchoprovocation tests can also be indicative.

Patients may also be getting breathless appropriately as they reach their physiological limit. This then is more an issue with the perception of the symptom rather than a physiological manifestation per-say. Symptoms in this case are always associated with exertion and often to a high degree (29).

Other causes of upper-airway obstruction can contribute to symptoms of dyspnoea. This could be laryngomalacia or subglottic stenosis (29) and there are even case reports of extrinsic compression in the airway presenting as dysfunctional breathing (30).

There may be a biomechanical issue such as scoliosis or pectus excavatum or carinatum, causing a restrictive respiratory defect, reducing the patients respiratory reserve and therefore contributing to dyspnea. These abnormalities can be associated with neuromuscular problems which can themselves be associated with hypoventilation and sometimes even symptoms of dyspnea as well (29).

More rarely there may be a problem with the cardiac rhythm or pump and some sort of either inherited or acquired cardiac disease associated with the symptom of dyspnea (29). Some specific pediatric examples of this include supraventricular tachycardia, cardiomyopathy, cyanotic and acyanotic structural congenital heart disease (31). Some pointers to this may include associated chest pain, peripheral oedema, a cardiac murmur, abnormalities on ECG and echocardiography. Rarer still, there may be a problem with the lung parenchyma itself such as interstitial lung disease. There are numerous causes of pediatric interstitial lung disease (32), but in our experience, the ones that most commonly present in the context of exercise related dyspnea are lung growth abnormalities often related to prematurity, or those associated with another primary systemic disease particularly autoimmune diseases.

### Examination

#### Breathing Pattern Assessment

A thorough examination of breathing pattern is required as a superficial examination (particularly when fully clothed and at rest) may appear normal. A detailed breathing pattern analysis is best performed by an expert physiotherapist and should be done whilst at rest and then during a provoked attack. Provocation in children is commonly through exercise but may also be perfumes or sprays. The breathing pattern analysis should be done through observation, palpation and using scanning techniques such as structured light plethysmography (SLP) (33) when the necessary equipment is available. SLP involves the projection of structured light, in the form of a checkerboard, on to the chest. The deformation of the checkers caused by breathing movements are digitally recorded and displayed graphically and numerically. Key aspects to assess (by whichever methods are available) include respiratory rate, ratio, dominance, route of breathing (at rest and during exertion) and sigh rate. Additional observations include stridor, voice changes, musculoskeletal imbalance, postural problems and core stability. Different types of abnormal breathing pattern in adults, as described earlier, include periodic deep sighing, thoracic dominant and thoraco-abdominal asynchrony (15, 34). Evidence is not yet available demonstrating whether these classifications of breathing pattern are also typical in children but most commonly the pediatric patient will present with a thoracic dominant pattern, increased RR, increased sigh rate and mouth breathing. Patterns observed may change dependent on the situation. Some children may present with a good breathing control in a stable or comfortable situation but may lose control in times of physical or mental stress (15).

Questionnaire based tools are useful both for assessment purposes but also as outcome measures for the efficacy of treatment when applied pre and post intervention. The most used questionnaire is the Nijmegen questionnaire (35). It wasn't designed for detecting DB as we recognize it now and is not validated for use in children. It can however be useful as a symptom score or evaluation of treatment as part of a multicomponent assessment (36).

Attempts have been made to develop other more targeted questionnaires. The SHAPE questionnaire (37, 38) was formulated to identify HVS in children and the Self Evaluation of Breathing Questionnaire (SEBQ) (39) to identify dysfunctional breathing in adults. Neither is therefore suitable for use in children with DB.

The most recent development is the Breathing Pattern Assessment Tool (BPAT) (40) which is an assessment instrument for detecting BPD. It has been trialed in adults with asthma, but work is needed to understand and validate its use in the pediatric population.

Questionnaires designed more specifically to distinguish/diagnose, monitor symptoms and measure quality of life in ILO are The Pittsburgh Vocal Cord Dysfunction Index (41),The VCD-5 (42), The VCDQ (43) and the SF-36v2 (44). These suffer with the same limitation as many of the BPD questionnaires, where they have not been validated in children.

### Investigations

Shortness of breath is the most common presentation of BPD and ILO. This is a symptom in many respiratory disorders and therefore DB should be considered when other conditions have been ruled out, or when other conditions are known but thought to be under control but the symptoms described seem to be disproportionate. As such investigations to rule out other causes should be ordered as appropriate. This may include a Chest Xray, ECG, Echocardiography, exhaled Nitric oxide levels, baseline spirometry with inspiratory loops and Beta 2 agonist reversibility, and bronchoprovocation studies.

#### Exercise Testing

If symptoms of DB are described to be exercise induced, then exercise testing should be performed. This may start with a simple exercise induced bronchoconstriction (EIB) test to exclude an asthma diagnosis, or, alternatively eucapnic voluntary hyperventilation (EVH) or other bronchoprovcation tests may be performed. If there is a high suspicion of EILO an exercise test with a flexible laryngoscopy may be performed.

Whilst some groups have reported exercise tests in adults which are specific to the sport in which the symptoms occur (e.g., cycling or swimming) (45), we have found all children are familiar with running and for the majority of clinical teams, a treadmill test will be the most feasible form of assessment. However, other exercise modalities (e.g., cycling) can be used when considering the context of the provoking symptoms with the individual patient. The exercise test can be performed with a flexible laryngoscope *in situ* during exercise, which provides the opportunity to visualize the larynx throughout the exercise cycle (46). This “continuous exercise laryngoscopy” has been referred to as the “gold standard” (6), however there are some limitations related to the availability and suitability of equipment in younger children and of being able to reach symptom provoking intensity whilst wearing the laryngoscope in children. We have found that pre and post exercise provocation laryngoscopy is well tolerated and can be sufficient to diagnose EILO within a full MDT assessment. It is important to note however, that in some cases, when symptoms resolve very quickly, false negatives will occur and continuous laryngoscopy with exercise would be the most suitable method to provide a definitive diagnosis.

The exercise test is performed on a treadmill (or other modality, dependent on the individual patient and what provokes symptoms) and should include a 12 lead ECG and continuous pulse oximeter measurements to eliminate any cardiac causes. Blood pressure measurements taken pre and post exercise can also be used to exclude post exercise hypotension. Patients are asked to perform a Borg score for breathlessness and leg tiredness pre and post exercise. The exercise test follows a similar protocol to a standard bronchoprovation protocol (47). The exercise starts with ~1 min of steady walking to acclimatize the patient to the treadmill, the treadmill settings are then adjusted to increase the gradient and speed to increase ventilation rapidly. Heart rate approaching 80% heart rate max can be used as a surrogate for ensuring rapid ventilation. In children, this is normally achieved with increasing the gradient of the treadmill (up to 20% in the more athletic children) more than the speed. In a bronchoprovocation test, the treadmill settings are adjusted to ensure the patient maintains an elevated heart rate for around 6 min, however in an exercise laryngoscopy test, the test should continue until symptoms occur. The onset of symptoms can vary in each patient, with some patients exhibiting symptoms at the onset of exercise, and others requiring high intensity and sustained exercise to provoke symptoms. It is recommended to continue exercising (with the patients consent) to symptom provocation. Once symptoms have commenced the upper airway is visualized via laryngoscopy (if not being continuously observed).

Spirometry with inspiratory loops should be performed pre and post exercise to investigate bronchoconstriction and any change in inspiratory loop which may indicate upper airway obstruction. If exercise induced bronchoconstriction is still suspected, spirometry should be performed at regular intervals up to 30 min post exercise as per standard bronchoprovocation protocols (47). Spirometry with asthma often shows obstruction (FEV_1_/FVC ratio below the lower limit of normal) with expiratory loop scooping and sometimes inspiratory loop flattening. With DB, spirometry is usually normal although there may be early termination of the expiratory loop and, with ILO specifically, inspiratory loop flattening is occasionally seen. These findings may be indicative but are not diagnostic (48).

#### Laryngoscopic Investigation

**Figure 4:**
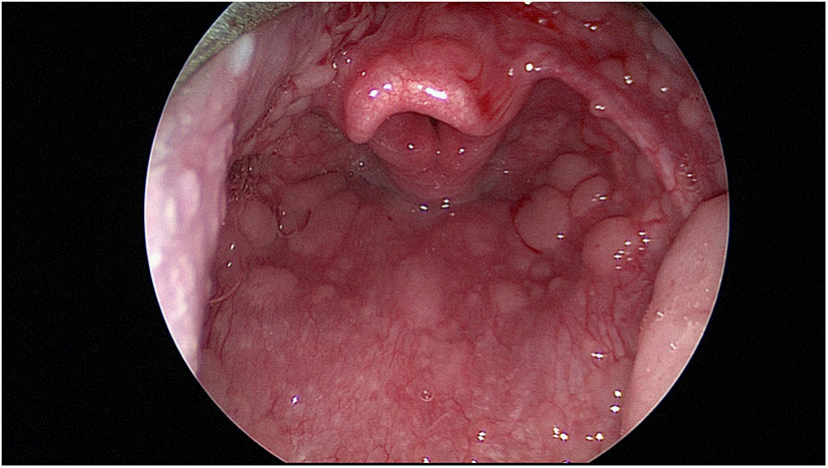
Laryngoscopic view showing unusually severe “cobblestone” appearance of the pharynx.

Flexible fiberoptic nasal laryngoscopy is performed at rest. If possible local anesthetic spray/decongestion is avoided but used if the child finds the procedure painful or distressing. An assessment can be made of the nasal airway and adenoids noting nasal disease such as rhinitis, polyps or adenoidal hypertrophy. A view of the larynx is then made. Pharyngeal changes such as “cobblestoning” may be seen (see Figure 4). This is a sign of inflammation and potentially of reflux (49). The position of the supraglottis, particularly the epiglottis, is noted. At this point erratic cordal movements during the breathing cycle may be noted even at rest.

The child is then exercised as described above, until the development of symptoms. At the point at which the symptoms are maximal, the treadmill is stopped and another nasal endoscopy procedure is performed (unless the provision for continuous laryngoscopy is available and indicated). At this point the same things are assessed particularly cordal movements in relation to the breathing cycle.

Cordal closure on inspiration is pathognomonic. Epiglottic prolapse or posterior glottic prolapse (also known as supraglottic closure) is also looked for and noted. In this acute phase an attempt may be made to assess effectiveness of maneuvers such as “nasal sniff” on cordal opening.

### Typical Findings of EILO

- Baseline spirometry is typically normal and baseline breathing pattern is typically abnormal (50)
- Abnormalities in their upper airway on pre-exercise nasal laryngoscopy, e.g., cobblestoning, which might support a diagnosis of reflux as an “enhancer”
- Audible stridor as the patient approaches maximal exercise and becomes symptomatic
- Post exercise nasal laryngoscopy will show indicative features as described above
- Symptoms disappear quickly on the cessation of exercise
- Post exercise spirometry is normal although inspiratory loops may be flattened.

## Management

### Medical

It is important to ensure any associated conditions like asthma, extra-esophageal reflux, rhinitis and allergy are treated appropriately and well controlled.

For asthma this will involve the appropriate use of asthma medication including inhaled short and long-acting B2 agonists, inhaled corticosteroids, leukotriene receptor antagonist, theophylline and anti IgE monoclonal antibodies.

For reflux this will involve lifestyle and dietary advice and use of antacids, H2 (histamine-2) blockers and proton pump inhibitors (PPIs) as appropriate.

For rhinitis this may involve the use of corticosteroid nasal sprays, antihistamine nasal sprays or saline nasal rinses (51).

For allergy this will involve the use of appropriate oral antihistamine therapy and in selected patients immunotherapy may also be appropriate (52).

### Therapeutic

**Figure 5:**
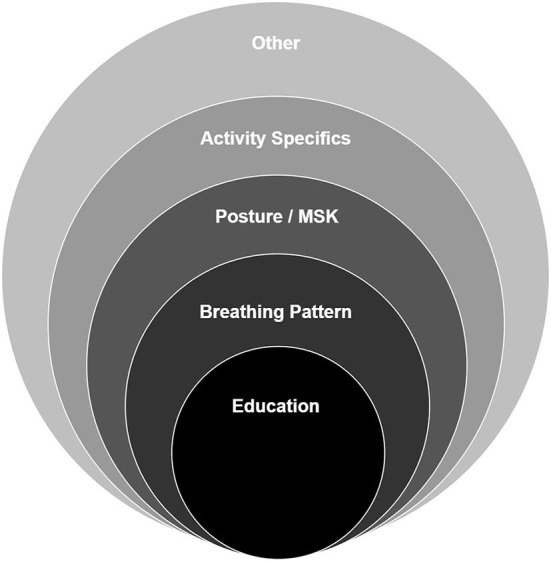
Typical framework for intervention used by a physiotherapist.

Most young people with dysfunctional breathing will require a course of non-pharmaceutical therapy to enable them to return to normal function. Therapy should be based on an individualized assessment followed by goal setting and treatment planning jointly agreed with the patient and their family. Interventions are most effective where a framework of approaches is used but should be individualized according to the assessment findings. Therapy is commonly provided by an experienced physiotherapist, speech and language therapist or psychologist depending on the dominant features of the DB presentation (i.e., the extent to which ILO and BPD contribute to the overall diagnosis of DB) and the specialist skills of the therapist. Some patients will benefit from input from more than one of these disciplines. Figure 5 shows a typical framework for intervention used by a physiotherapist.

Education is key to a successful outcome. The education package needs to be tailored to the child's age/ability and includes information about the nature of the condition and promotes a basic understanding of the anatomy, physiology and mechanics of breathing. This allows the child to understand their situation, reduce their fear (53) and start to feel in control of what is happening. It also provides a rationale for active therapy participation (54). Depending on the type of BD and any co-morbidities present, education may also be aimed at helping the child to differentiate between their symptoms (e.g., DB vs, asthma) and understand when to apply therapeutic techniques or use medication.

Education is also important for parents, teachers, coaches and other professionals to enable them to support the child's therapeutic journey. It is useful to use a quiz to gauge the child's knowledge and understanding and to ensure the therapist and child are working from a common position.

Breathing retraining is the primary active intervention and is aimed at redressing the abnormal or altered breathing pattern (55). It is commonly addressed through the teaching of diaphragmatic breathing, first in more manageable positions such as crook lying with progression through to more demanding and functional positions. Poor posture is frequently associated with DB and the method for addressing it depends on the underlying cause. Approaches range from simple awareness of the need for good posture and what this constitutes, through to stretching, strengthening and core stability exercises.

Activity specific interventions are required to ensure that patients reach their own specific goals. The interventions will vary according to the goal but an example would be a brass or woodwind musician who is unable to complete their solo may need to work on preparation of breathing and posture for playing or supporting the column of air to sustain the length, volume or stability of notes.

There are also a range of other interventions which may be required depending on the individual problems identified. These can include nasal rinsing, airway clearance techniques, education to improve inhaler technique and support to improve compliance, and relaxation. Sleep onset delay is commonly reported by young people with DB. It commonly resolves with improvement in breathing pattern but can also be addressed through improved sleep hygiene or the use of acupressure for stimulating the release of melatonin. Inspiratory muscle training (IMT) is sometimes used for the treatment of DB in adolescent and adult patients (56). There is however no evidence for the use of IMT in younger children with DB who are otherwise fit and healthy and little evidence in those with co-morbidities such as asthma (57). There is concern amongst therapists that IMT may have a negative impact on the quality of the breathing pattern but there may be potential for benefit in young people participating in high level sport, particularly in the latter stages of their treatment program. This is an area that will profit from further research.

Speech and language therapy is indicated for those presenting with a notable upper airway component. The focus is primarily on management of the upper airway and approaches can be divided in to short and long term strategies. The reduction of irritants through the practice of good upper airway hygiene and awareness/management of triggers can reduce sensitivity and hyper-responsiveness (26, 58), whilst acute symptoms can be controlled by activities that promote and maintain vocal cord abduction such as sniffs and vocal fricatives (59). Longer term retraining of glottic and supraglottic activity can be achieved through laryngeal aperture control (with or without the addition of therapeutic laryngoscopy) and use of the Accent method (60, 61). Therapy can also be optimized by effective management of co-morbidities such as using voice therapy techniques and relaxation.

The role of psychology varies in this condition depending on the type of presentation. Those cases where anxiety or performance related stressors play a significant part will benefit from the involvement of an experienced clinical or sports psychologist. Most other cases will also benefit from some degree of psychological support, but it may be appropriate for this to be provided by the clinical team or through counselors or other support structures at schools or clubs. Psychological support is also important to help young people understand the interaction between their physical and mental health.

With appropriate breathing therapy, long term outcomes in children and young people are good, and recurrence is rare (62, 63).

### Surgical

If there is a structural abnormality, then surgery may have a role. Adenoidal hypertrophy and rhinitis are generally treated with intranasal steroid therapy in the first instance. Adenoidectomy and turbinate reduction may be helpful.

If supraglottic EILO is present, supraglottoplasty or epiglottopexy may be of benefit. These operations are relatively morbid and therefore surgery for paroxysmal supraglottic collapse should be considered as a “last resort.” In our experience, surgery is not commonly required but other centers working with adolescents and adults have demonstrated encouraging results for patients with difficult to treat presentations (64–67).

## Conclusion

Dysfunctional breathing describes a complex relationship between breathing pattern disorders and inducible laryngeal obstruction. The diagnostic pathway for dysfunctional breathing is driven by detailed history taking which will indicate the appropriate tests required to distinguish the form of DB present and help rule out other potential causes of symptoms. An individualized treatment program based on expert assessment and personalized goals will then enable the young person to successfully return to normal function. It is expected that accurate and timely diagnosis of all types of dysfunctional breathing leads to good outcomes, and with a combination of the management options described above, patients will return to all normal daily activities and the degree of sport commensurate with their level of physical fitness and skill.

## Author Contributions

The manuscript was written by the multi-disciplinary team working in the ExL (Exercise laryngoscopy) clinic, Sheffield, UK. The team consists of a Physiotherapist (NB), Physiologist (JK), ENT Surgeon (RT) and Respiratory Consultant (KU). NB coordinated the work. All authors made a substantial contribution to the conception of the work, original ideas and drafting of the manuscript, with each author contributing primarily to the area within their expertise. All authors contributed to the article and approved the submitted version.

## Conflict of Interest

The authors declare that the research was conducted in the absence of any commercial or financial relationships that could be construed as a potential conflict of interest.
